# Evaluation of papillary myocardial infarction: incremental value of a short time inversion vs standard late enhancement imaging

**DOI:** 10.1186/1532-429X-13-S1-P80

**Published:** 2011-02-02

**Authors:** Annachiara Aldrovandi, Stijin PJ De Ridder, Oliver Strohm, Myra Cocker, Matthias Friedrich

**Affiliations:** 1 Department od Cardiology, Azienda Ospedaliero-universitaria di Parma, Parma, Italy; 2 Department of Cardiology, Academic Hospital Maastricht, Maastricht,, Netherlands; 3 Department of Cardiac Sciences and Radiology, Stephenson Cardiovascular MR Centre at the Libin Cardiovascular Institute of Alberta, Calgary, AB, Canada

## Introduction

Papillary muscle involvement in acute myocardial infarction (MI) may be associated with new-onset mitral regurgitation and ventricular arrhythmias. Late gadolinium enhancement (LGE) cardiovascular magnetic resonance (CMR) imaging can detect and quantify myocardial scar. The detection of papillary muscle involvement however may be challenged by difficulties discriminating the affected muscle from the bright blood signal

## Purpose

The aim of our study was to evaluate the incremental value of LGE CMR imaging using an inversion recovery (IR)-GRE with short inversion time over standard LGE CMR imaging in identifying papillary muscle involvement in patients with previous myocardial infarction.

## Methods

Fifty-six patients with chronic myocardial infarction were studied using a standard IR- GRE LGE sequence with an individually adjusted inversion time (TI) to null the signal intensity of normal myocardium and with a 3D IR-GRE with a short inversion time (<160 ms). Signal-to-noise (SNR) and contrast-to-noise ratios and the frequency of papillary muscle infarction were determined. Furthermore, image quality and subjective distinction of the infarction (sharpness) were quantified using a grading system.

## Results

The short TI LGE sequence detected a significant higher number of papillary muscle infarction compared to standard LGE sequence (19/54 versus 15/54 respectively). Moreover, in these images papillary muscle infarction was appeared with more sharpness (84.2% vs 53.3%) The contrast-to-noise ratio was higher between infarcted myocardium and blood (77.9±60 vs 19.3±16, p<0.001) and between papillary muscle infarction and blood (69.4±51 vs 39.4±26 respectively, p=0.0157).

## Conclusions

In patients with myocardial infarction, LGE CMR imaging using short inversion times more sensitively detects papillary muscle infarction when compared with standard LGE imaging. Therefore, the additional use of short TI sequences may be useful for verifying or excluding papillary muscle involvement in patients with myocardial infarction.

